# Prevalence of Missing Data in the National Cancer Database and Association With Overall Survival

**DOI:** 10.1001/jamanetworkopen.2021.1793

**Published:** 2021-03-23

**Authors:** Daniel X. Yang, Rohan Khera, Joseph A. Miccio, Vikram Jairam, Enoch Chang, James B. Yu, Henry S. Park, Harlan M. Krumholz, Sanjay Aneja

**Affiliations:** 1 Department of Therapeutic Radiology, Yale School of Medicine, New Haven, Connecticut; 2 Department of Internal Medicine, Yale School of Medicine, New Haven, Connecticut; 3 Center for Outcomes Research and Evaluation, Yale School of Medicine, New Haven, Connecticut

## Abstract

**Importance:**

Cancer registries are important real-world data sources consisting of data abstraction from the medical record; however, patients with unknown or missing data are underrepresented in studies that use such data sources.

**Objective:**

To assess the prevalence of missing data and its association with overall survival among patients with cancer.

**Design, Setting, and Participants:**

In this retrospective cohort study, all variables within the National Cancer Database were reviewed for missing or unknown values for patients with the 3 most common cancers in the US who received diagnoses from January 1, 2006, to December 31, 2015. The prevalence of patient records with missing data and the association with overall survival were assessed. Data analysis was performed from February to August 2020.

**Exposures:**

Any missing data field within a patient record among 63 variables of interest from more than 130 total variables in the National Cancer Database.

**Main Outcomes and Measures:**

Prevalence of missing data in the medical records of patients with cancer and associated 2-year overall survival.

**Results:**

A total of 1 198 749 patients with non–small cell lung cancer (mean [SD] age, 68.5 [10.9] years; 628 811 men [52.5%]), 2 120 775 patients with breast cancer (mean [SD] age, 61.0 [13.3] years; 2 101 758 women [99.1%]), and 1 158 635 patients with prostate cancer (mean [SD] age, 65.2 [9.0] years; 100% men) were included in the analysis. Among those with non–small cell lung cancer, 851 295 patients (71.0%) were missing data for variables of interest; 2-year overall survival was 33.2% for patients with missing data and 51.6% for patients with complete data (*P* < .001). Among those with breast cancer, 1 161 096 patients (54.7%) were missing data for variables of interest; 2-year overall survival was 93.2% for patients with missing data and 93.9% for patients with complete data (*P* < .001). Among those with prostate cancer, 460 167 patients (39.7%) were missing data for variables of interest; 2-year overall survival was 91.0% for patients with missing data and 95.6% for patients with complete data (*P* < .001).

**Conclusions and Relevance:**

This study found that within a large cancer registry–based real-world data source, there was a high prevalence of missing data that were unable to be ascertained from the medical record. The prevalence of missing data among patients with cancer was associated with heterogeneous differences in overall survival. Improvements in documentation and data quality are necessary to make optimal use of real-world data for clinical advancements.

## Key Points

**Question:**

What is the prevalence of missing data in the medical record, and is this prevalence associated with outcome estimation for patients with cancer?

**Findings:**

In this cohort study of more than 4 million patients with cancer using abstracted medical records from the National Cancer Database, a high prevalence of missing data for patients with the 3 most common cancers in the US was found. Patients with missing data had worse overall survival than those with complete data.

**Meaning:**

The study’s findings suggest substantial gaps in documentation and data capture via the medical record for patients with cancer.

## Teaser

This cohort study examines real-world data in the National Cancer Database to assess the prevalence of missing data and the association between missing data and overall survival among patients with the 3 most common types of cancer in the US.

## Introduction

Real-world evidence derived from real-world data (RWD) has substantial potential to accelerate innovation within oncology. Real-world data sources, which include routinely collected information on patient health status and/or the delivery of health care,^1^ are becoming increasingly relevant because of the high cost and slow pace of randomized clinical trials as well as the growth of almost real-time access to electronic health records and other digital sources of comprehensive health-associated data. Real-world data sources may represent a flexible and cost-effective way to investigate clinical interventions and can supplement data from clinical trials. Within the oncology field, investments have been made to develop RWD sources for clinical evidence generation, both at the national level and within professional societies.^2,3,4,5^

Cancer registries have long been established as important sources of RWD that can generate insights spanning the epidemiologic characteristics of cancer and to the comparative-effectiveness analysis of therapies.^2,6^ Data quality, including the completeness of data elements, is a major consideration when working with registries to generate clinical insights. This issue is particularly germane given emerging data suggesting that treatment-associated survival outcomes using registries vs similar randomized clinical trials are not concordant.^7,8,9^ There is a need to assess the quality of clinical data generated from registries and other RWD sources and to examine whether these sources have adhered to best data practices. Of note, cancer registries rely on trained tumor registrars to abstract and record data from the patient medical record. Lack of high-quality documentation within the medical record can produce incompletely abstracted data elements and therefore lead to unknown or missing data values within cancer registries.^10,11,12^

While there are a variety of methods to account for missing data, patients with unknown values are likely underrepresented in RWD studies, as it is common practice to exclude patients without complete information for variables used in cohort construction.^13,14,15,16^ However, because missing data within registries are surrogates for a lack of high-quality documentation, such data may not be missing completely at random, and the exclusion of patients with missing data may introduce substantial bias. In addition, missing data are relevant to clinical care, as they may reflect missing clinical data, such as cancer stage, that are important and are often used to guide treatment selection. Systematic evaluation of missing documentation among patients with cancer may elucidate the areas in which investments can be made to capture more complete data.^17^

In this study, we aimed to characterize the outcomes associated with unknown documentation across multiple cancer types within a large national cancer registry. We specifically examined the prevalence of missing data among patients with the 3 most common cancer types in the US and assessed whether the characteristics and overall survival of patients with missing data were comparable with those with complete data.

## Methods

We examined the prevalence of patient records with missing data and the association with overall survival among patients with cancer in a large cancer registry that is commonly used for comparative-effectiveness studies of the 3 most common cancers in the US (non–small cell lung cancer [NSCLC], breast cancer, and prostate cancer). We compared overall survival differences between patients who had complete vs missing data. This study was approved by the Yale University Human Investigation Committee and was granted an exemption of informed consent because deidentified patient information was used. This study followed the Strengthening the Reporting of Observational Studies in Epidemiology (STROBE) reporting guideline for cohort studies.

The National Cancer Database (NCDB) was established in the 1980s and is jointly sponsored by the American College of Surgeons Commission on Cancer and the American Cancer Society.^18^ More than 130 variables are included in the NCDB participant user file, capturing a range of facility and patient information, tumor characteristics, treatment information, and cancer outcomes that are abstracted from medical records by trained tumor registrars.^19,20^ Additional details regarding the NCDB are available in eMethods in the Supplement.

We identified 96 variables in the NCDB that were used for all years of diagnosis and disease sites included in our analysis. From those, we identified variables that were missing data in at least 1 patient record. Missing data were defined as either empty data fields or unknown data entries for a variable included in the database. Two clinical oncologists (D.X.Y. and S.A.) reviewed all variables and excluded those for which empty data fields were allowed by the NCDB data dictionary and thus may not have reflected incomplete clinical documentation. A total of 63 final variables of interest were identified to compare patients with complete vs missing data (Table 1; eMethods and eTable 1 in the Supplement).

**Table 1.** Distribution of Variable Types Among Study Population

Variable type	Variables, No. (%)
All variables	
Total, No.	96
Demographic	22 (22.9)
Cancer identification	11 (11.5)
Cancer stage	18 (18.8)
Cancer treatment	41 (42.7)
Outcomes	4 (4.2)
Variables with any missing data	
Total, No.	82
Demographic	14 (17.1)
Cancer identification	6 (7.3)
Cancer stage	18 (22.0)
Cancer treatment	41 (50.0)
Outcomes	3 (3.7)
Variables of interest	
Total, No.	63
Demographic	14 (22.2)
Cancer identification	6 (9.5)
Cancer stage	13 (20.6)
Cancer treatment	30 (47.6)

In the NCDB participant user file, we identified patients with NSCLC, breast cancer, and prostate cancer who received cancer diagnoses from January 1, 2006, to December 31, 2015. Because of changes in data coding rules that introduced new variables and a lack of survival information for the most recent year of diagnosis, we excluded patients who received diagnoses in 2016. Given changes in data reporting standards and completeness over time, we examined cancer cases diagnosed in the most recent 10 years before 2016. The follow-up period investigated for overall survival included all available follow-up events recorded in the database. The outcomes associated with missing data were assessed by cancer stage, as defined by the NCDB analytic staging group.^21^

### Statistical Analysis

We calculated the percentage of patients with missing or unknown values in any 1 of the 63 variables of interest. We used standard descriptive statistics, a χ^2^ test, and a Wilcoxon rank sum test to identify differences in patient, tumor, and treatment characteristics between those with missing vs complete data. A patient record was excluded for comparison of patient, tumor, and treatment characteristics if the record had a missing value in the variable being compared (tabulation shown in eTable 2 in the Supplement). We used Kaplan-Meier estimates to compare overall survival between patients with missing vs complete data. The primary outcome was the prevalence of missing data and its association with 2-year overall survival. A secondary analysis stratifying results by cancer stage and treatment was also performed. A log-rank test was used to identify statistically significant differences in overall survival. We used *P* < .05 as the a priori threshold for statistical significance. Hypothesis tests were unpaired. Bonferroni correction was used to account for multiple comparisons. The significance threshold for the subgroup analysis was *P* < .004 after adjustment.

For the sensitivity analysis, we tested an alternative approach for identifying variables by including data that were missing in 1% to 20% of patient records. This range was determined a priori because records with less than 1% of missing data are likely to have few consequences for outcomes of RWD studies, and a large percentage of missing data is more likely to be reflective of explainable differences in coding rules rather than a lack of documentation quality. Different percentage thresholds of missing data were also tested (eFigure 9 in the Supplement). To explore the relative importance of missing data for each individual variable of interest, we also performed a univariable Cox regression analysis using a missing indicator for each variable of interest (eTable 3 in the Supplement).

Statistical analysis was performed using Stata software, version 16 (StataCorp). The code used is available through a public GitHub repository.^22^ Data were analyzed from February to August 2020.

## Results

Of the 96 data elements included for analysis, 22 variables (22.9%) pertained to demographic characteristics, 11 variables (11.5%) to tumor characteristics, 18 variables (18.8%) to cancer stage, 41 variables (42.7%) to treatment, and 4 variables (4.2%) to outcomes. After limiting the analysis to 63 variables of interest, 14 demographic variables (22.2%), 6 tumor characteristic variables (9.5%), 13 cancer stage variables (20.6%), and 30 treatment variables (47.6%) were included (Table 1).

A total of 1 198 749 patients had NSCLC (mean [SD] age, 68.5 [10.9] years; 628 811 men [52.5%]; 1 024 372 White [85.5%]), 2 120 775 patients had breast cancer (mean [SD] age, 61.0 [13.3] years; 2 101 758 women [99.1%]; 1 761 964 White [83.1%]), and 1 158 635 patients had prostate cancer (mean [SD] age, 65.2 [9.0] years; 1 158 635 men [100%]; 940 943 White [81.2%]) (Table 2). With regard to cancer stage, most patients with NSCLC had stage IV disease (458 371 patients [38.2%]), most patients with breast cancer had stage I disease (850 058 patients [40.1%]), and most patients with prostate cancer had stage II disease (760 555 patients [65.6%]). Among those with NSCLC, 543 481 patients (45.3%) had lymph node involvement, and 453 069 patients (37.8%) had distant metastasis. Of those with breast cancer, 444 822 patients (21.0%) had lymph node involvement, and 86 191 patients (4.1%) had distant metastasis. Among those with prostate cancer, 37 535 patients (3.2%) had lymph node involvement, and 54 997 patients (4.7%) had distant metastasis.

**Table 2.** Patient, Disease, and Treatment Characteristics

Characteristic	Patients, No. (%)^a^^,^^b^	
	Complete data	Missing data
**Non–small cell lung cancer**		
Total patients, No.	347 454	851 295
Age at diagnosis, median (IQR), y	69 (62-76)	69 (61-77)
Sex		
Male	177 594 (51.1)	451 217 (53.0)
Female	169 860 (48.9)	400 078 (47.0)
Race		
White	303 607 (87.4)	720 765 (84.7)
Black	34 565 (9.9)	95 560 (11.2)
Other^c^	9282 (2.7)	25 802 (3.0)
Ethnicity		
Non-Hispanic	338 785 (97.5)	758 913 (89.1)
Hispanic	8669 (2.5)	25 102 (2.9)
Charlson-Deyo comorbidity score		
0	184 687 (53.2)	503 684 (59.2)
1	108 556 (31.2)	229 207 (26.9)
2	38 916 (11.2)	83 537 (9.8)
≥3	15 295 (4.4)	34 867 (4.1)
Insurance		
Not insured	8818 (2.5)	27 945 (3.3)
Private	92 017 (26.5)	226 175 (26.6)
Medicaid	19 886 (5.7)	53 265 (6.3)
Medicare	222 107 (63.9)	506 860 (59.5)
Other government	4626 (1.3)	13 691 (1.6)
Facility type		
Community	240 682 (69.3)	571 663 (67.2)
Academic	106 772 (30.7)	271 994 (32.0)
Tumor		
Year of diagnosis, median (IQR)	2011 (2009-2013)	2010 (2008-2013)
Overall stage		
I	145 393 (41.8)	171 141 (20.1)
II	44 488 (12.8)	55 601 (6.5)
III	74 441 (21.4)	174 460 (20.5)
IV	83 073 (23.9)	375 298 (44.1)
Tumor size, cm		
≤3	167 184 (48.1)	278 361 (32.7)
>3	179 778 (51.7)	347 749 (40.8)
Lymph nodes involved		
No	197 933 (57.0)	287 971 (33.8)
Yes	138 977 (40.0)	404 504 (47.5)
Distant metastasis		
No	263 796 (75.9)	445 966 (52.4)
Yes	83 658 (24.1)	369 411 (43.4)
Treatment		
Surgery (primary site)		
No	174 754 (50.3)	669 039 (78.6)
Yes	172 700 (49.7)	178 671 (21.0)
Radiotherapy		
No	223 946 (64.5)	481 005 (56.5)
Yes	123 508 (35.5)	359 919 (42.3)
Chemotherapy		
No	207 763 (59.8)	434 274 (51.0)
Yes	139 691 (40.2)	376 777 (44.3)
**Breast cancer**		
Total patients, No.	959 679	1 161 096
Age at diagnosis, median (IQR), y	62 (53-72)	59 (49-70)
Sex		
Male	8552 (0.9)	10 465 (0.9)
Female	951 127 (99.1)	1 150 631 (99.1)
Race		
White	814 602 (84.9)	947 362 (81.6)
Black	105 594 (11.0)	137 369 (11.8)
Other^c^	39 483 (4.1)	53 425 (4.6)
Ethnicity		
Non-Hispanic	915 866 (95.4)	982 844 (84.6)
Hispanic	43 813 (4.6)	66 997 (5.8)
Charlson-Deyo comorbidity score		
0	786 312 (81.9)	997 133 (85.9)
1	137 187 (14.3)	131 158 (11.3)
2	27 511 (2.9)	24 880 (2.1)
≥3	8669 (0.9)	7925 (0.7)
Insurance		
Not insured	17 384 (1.8)	25 447 (2.2)
Private	486 495 (50.7)	626 116 (53.9)
Medicaid	53 951 (5.6)	70 871 (6.1)
Medicare	392 685 (40.9)	388 308 (33.4)
Other government	9164 (1.0)	11 747 (1.0)
Facility type		
Community	684 570 (71.3)	725 684 (62.5)
Academic	275 109 (28.7)	341 691 (29.4)
Tumor		
Year of diagnosis, median (IQR)	2012 (2009-2014)	2010 (2008-2013)
Overall stage		
0 (DCIS)	133 409 (13.9)	294 752 (25.4)
I	459 031 (47.8)	391 027 (33.7)
II	258 213 (26.9)	254 836 (21.9)
III	78 254 (8.2)	97 157 (8.4)
IV	30 454 (3.2)	51 889 (4.5)
Tumor size, cm		
≤2	629 447 (65.6)	610 410 (52.6)
>2	327 146 (34.1)	344 006 (29.6)
Lymph nodes involved		
No	731 333 (76.2)	760 552 (65.5)
Yes	214 178 (22.3)	230 644 (19.9)
Distant metastasis		
No	926 319 (96.5)	1 030 431 (88.7)
Yes	33 042 (3.4)	53 149 (4.6)
Treatment		
Surgery (primary site)		
No	46 302 (4.8)	109 849 (9.5)
Yes	913 377 (95.2)	1 046 754 (90.2)
Radiotherapy		
No	433 200 (45.1)	566 736 (48.8)
Yes	526 479 (54.9)	572 478 (49.3)
Chemotherapy		
No	634 319 (66.1)	697 497 (60.1)
Yes	325 360 (33.9)	395 557 (34.1)
Hormonal therapy		
No	347 616 (36.2)	487 554 (42.0)
Yes	612 063 (63.8)	578 864 (49.9)
**Prostate cancer**		
Total patients, No.	698 468	460 167
Age at diagnosis, median (IQR), y	65 (59-71)	65 (59-72)
Sex		
Male	698 468 (100.0)	460 167 (100.0)
Race		
White	579 894 (83.0)	361 049 (78.5)
Black	99 417 (14.2)	67 160 (14.6)
Other^c^	19 157 (2.7)	13 501 (2.9)
Ethnicity		
Non-Hispanic	669 071 (95.8)	366 527 (79.7)
Hispanic	29 397 (4.2)	20 141 (4.4)
Charlson-Deyo comorbidity score		
0	573 655 (82.1)	379 345 (82.4)
1	101 891 (14.6)	64 092 (13.9)
2	17 408 (2.5)	12 523 (2.7)
≥3	5514 (0.8)	4207 (0.9)
Insurance		
Not insured	11 414 (1.6)	9344 (2.0)
Private	337 278 (48.3)	205 477 (44.7)
Medicaid	17 389 (2.5)	12 835 (2.8)
Medicare	318 328 (45.6)	201 474 (43.8)
Other government	14 059 (2.0)	7415 (1.6)
Facility type		
Community	434 953 (62.3)	278 141 (60.4)
Academic	263 515 (37.7)	181 155 (39.4)
Tumor		
Year of diagnosis, median (IQR)	2010 (2008-2013)	2010 (2007-2012)
Overall stage		
I	96 492 (13.8)	48 900 (10.6)
II	493 798 (70.7)	266 757 (58.0)
III	73 637 (10.5)	43 243 (9.4)
IV	34 503 (4.9)	44 650 (9.7)
Lymph node involvement		
No	650 476 (93.1)	337 102 (73.3)
Yes	18 464 (2.6)	19 071 (4.1)
Distant metastasis		
No	677 567 (97.0)	383 731 (83.4)
Yes	20 862 (3.0)	34 135 (7.4)
Treatment		
Surgery (primary site)		
No	314 879 (45.1)	207 399 (45.1)
Yes	383 589 (54.9)	249 492 (54.2)
Radiotherapy		
No	446 325 (63.9)	303 962 (66.1)
Yes	252 143 (36.1)	145 409 (31.6)
Chemotherapy		
No	694 105 (99.4)	417 776 (90.8)
Yes	4363 (0.6)	5967 (1.3)
Hormonal therapy		
No	550 765 (78.9)	323 474 (70.3)
Yes	147 703 (21.1)	96 039 (20.9)

Abbreviations: DCIS, ductal carcinoma in situ; IQR, interquartile range.

^a^ The numbers of patient records with missing or unavailable data for each category are available in eTable 2 in the Supplement.

^b^ *P* < .001 for comparisons in all categories with the exception of sex (*P* = .43).

^c^ A large number of race categories are recorded in the National Cancer Database. Therefore, consistent with a previous study using data from the National Cancer Database,^23^ patients of non-White and non-Black races were recoded into the *other* category.

Differences were found in demographic characteristics, cancer stage, and treatments received between patients with complete and missing data. Among 347 454 patients with NSCLC who had complete data, 34 565 patients (9.9%) were Black, 8669 patients (2.5%) were Hispanic, 184 687 patients (53.2%) had a Charlson-Deyo comorbidity score of 0, 83 073 patients (23.9%) had stage IV disease, and 172 700 patients (49.7%) underwent surgery at the primary tumor site. Among 851 295 patients with NSCLC who had missing data, 95 560 patients (11.2%) were Black, 25 102 patients (2.9%) were Hispanic, 503 684 patients (59.2%) had a Charlson-Deyo comorbidity score of 0, 375 298 patients (44.1%) had stage IV disease, and 178 671 patients (21.0%) underwent surgery at the primary tumor site. Among 959 679 patients with breast cancer who had complete data, 105 594 patients (11.0%) were Black, 43 813 patients (4.6%) were Hispanic, 786 312 patients (81.9%) had a Charlson-Deyo comorbidity score of 0, 30 454 patients (3.2%) had stage IV disease, and 913 377 patients (95.2%) underwent surgery at the primary tumor site. Among 1 161 096 patients with breast cancer who were missing data, 137 369 patients (11.8%) were Black, 66 997 patients (5.8%) were Hispanic, 997 133 patients (85.9%) had a Charlson-Deyo comorbidity score of 0, 51 889 patients (4.5%) had stage IV disease, and 1 046 754 patients (90.2%) underwent surgery at the primary tumor site. Among 698 468 patients with prostate cancer who had complete data, 99 417 patients (14.2%) were Black, 29 397 patients (4.2%) were Hispanic, 573 655 patients (82.1%) had a Charlson-Deyo comorbidity score of 0, 34 503 patients (4.9%) had stage IV disease, and 383 589 patients (54.9%) underwent surgery at the primary tumor site. Among 460 167 patients with prostate cancer who had missing data, 67 160 patients (14.6%) were Black, 20 141 patients (4.4%) were Hispanic, 379 345 patients (82.4%) had a Charlson-Deyo comorbidity score of 0, 44 650 patients (9.7%) had stage IV disease, and 249 492 patients (54.2%) underwent surgery at the primary tumor site (Table 2).

### Missing Data and Overall Survival

**Figure 1.**
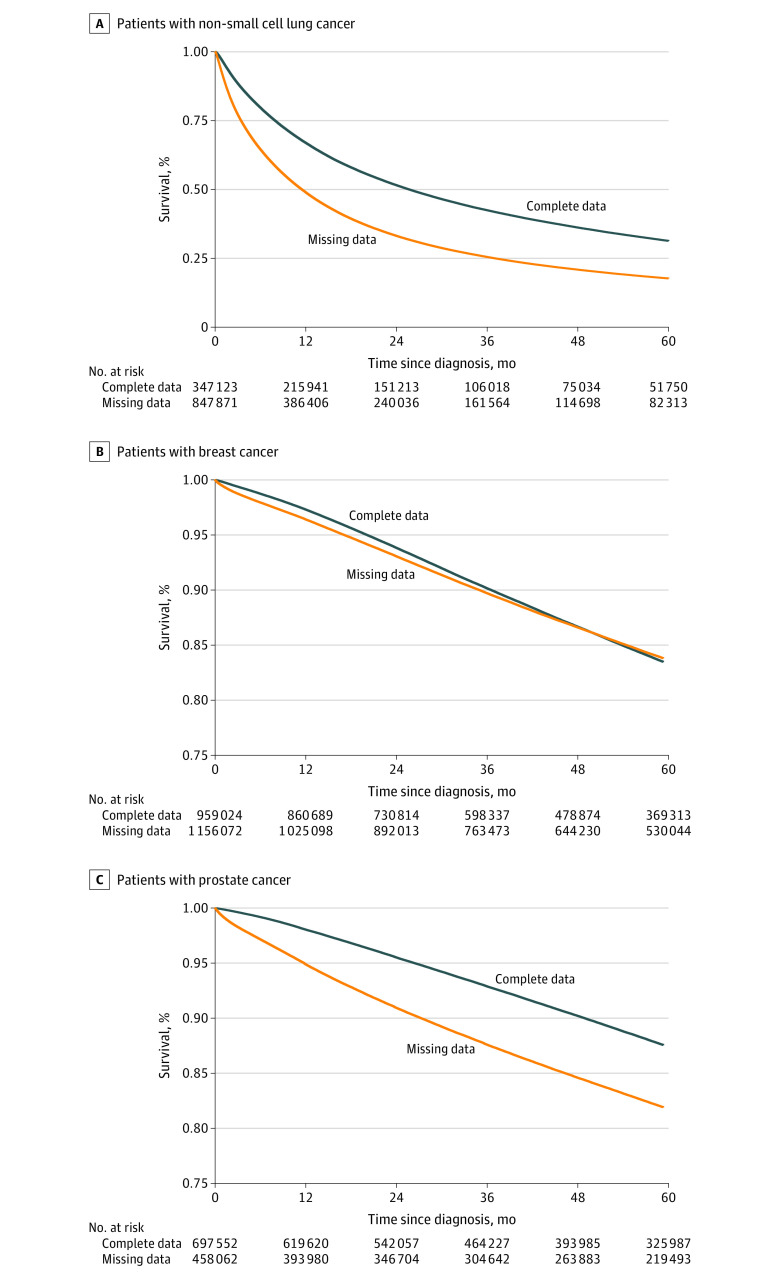
Overall Survival of Patients With Non–Small Cell Lung Cancer, Breast Cancer, and Prostate Cancer by Missingness of Data for Variables of Interest A, Patients with non–small cell lung cancer. B, Patients with breast cancer. C, Patients with prostate cancer.

Among those with NSCLC, 851 295 patients (71.0%) had missing data, and 347 454 patients (29.0%) had complete data; 2-year overall survival was 33.2% for patients with missing data and 51.6% for patients with complete data (*P* < .001) (Table 3; Figure 1). Among those with breast cancer, 1 161 096 patients (54.7%) had missing data, and 959 679 patients (45.3%) had complete data; 2-year overall survival was 93.2% for patients with missing data and 93.9% for patients with complete data (*P* < .001). Among those with prostate cancer, 460 167 patients (39.7%) had missing data, and 698 468 patients (60.3%) had complete data; 2-year overall survival was 91.0% for patients with missing data and 95.6% for patients with complete data (*P* < .001). These findings equate to an absolute 2-year overall survival difference of 18.4% for patients with NSCLC, 0.7% for patients with breast cancer, and 4.6% for patients with prostate cancer (Figure 1).

**Table 3.** Patients With Missing Data for at Least 1 Variable

Variable	Patients with missing data, No. (%)		
	Non–small cell lung cancer (n = 1 198 749)	Breast cancer (n = 2 120 775)	Prostate cancer (n = 1 158 635)
Any	851 295 (71.0)	1 161 096 (54.7)	460 167 (39.7)
Demographic	155 917 (13.0)	344 666 (16.3)	161 498 (13.9)
Cancer identification	560 754 (46.8)	284 281 (13.4)	93 111 (8.0)
Cancer stage	420 934 (35.1)	620 313 (29.2)	198 320 (17.1)
Cancer treatment	192 075 (16.0)	408 269 (19.3)	148 644 (12.8)

**Figure 2.**
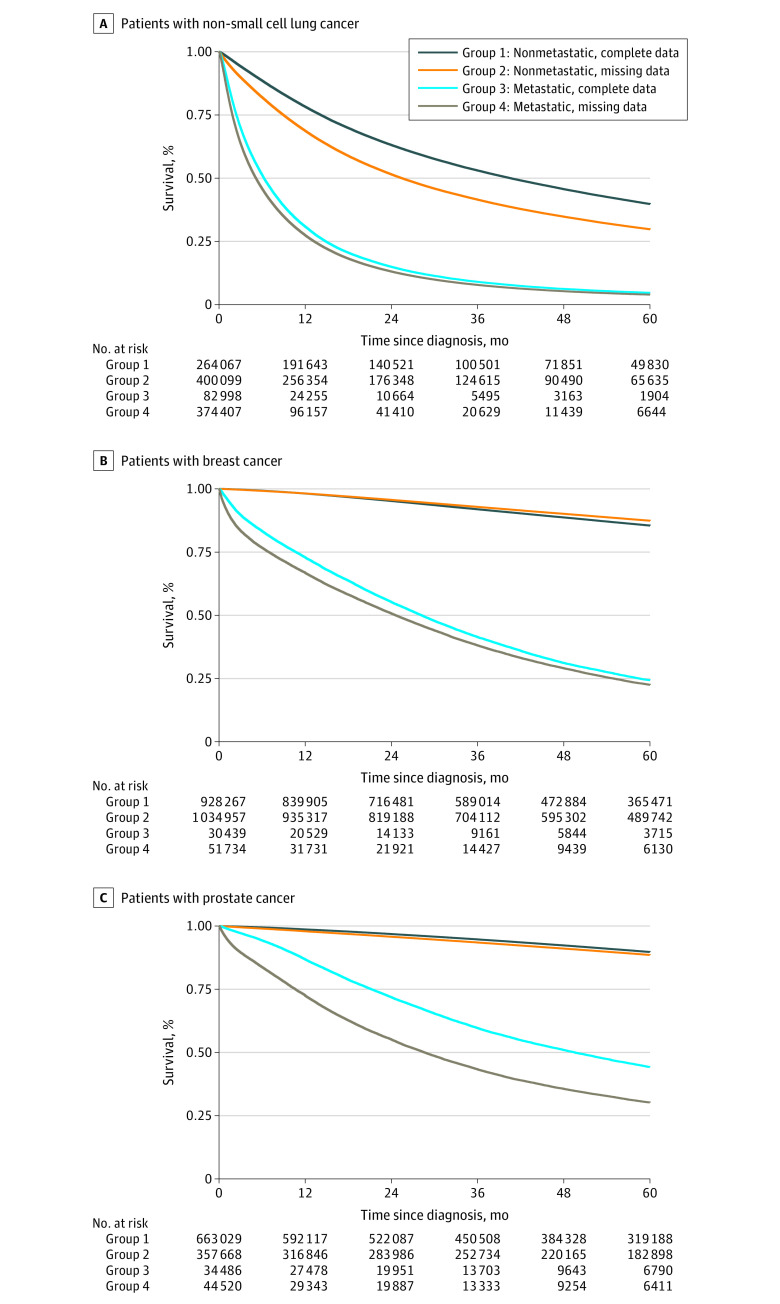
Overall Survival of Patients With Metastatic and Nonmetastatic Non–Small Cell Lung Cancer, Breast Cancer, and Prostate Cancer by Missingness of Data for Variables of Interest A, Patients with non–small cell lung cancer. B, Patients with breast cancer. C, Patients with prostate cancer.

Overall survival differences remained among patients with metastatic disease when stratified by cancer stage. Among those with nonmetastatic cancer, the absolute survival differences were smaller for patients with breast cancer (0.4%) and prostate cancer (1.1%) compared with survival differences of 4.5% for breast cancer and 16.7% for prostate cancer among patients with metastatic disease (*P* < .001 for both comparisons) (Figure 2). Among patients with metastatic NSCLC, 2-year overall survival was 13.1% for those with missing data and 15.0% for those with complete data (*P* < .001), whereas among patients with nonmetastatic NSCLC, 2-year overall survival was 51.5% for those with missing data and 63.2% for those with complete data (*P* < .001). Results of the secondary analysis of overall survival stratified by cancer stage are shown in eFigure 1, eFigure 2, and eFigure 3 in the Supplement, and results of overall survival stratified by receipt of surgery, radiotherapy, or chemotherapy are shown in eFigure 4 in the Supplement.

Temporal changes were observed in the proportion of missing data from 2006 to 2015. During this period, the percentage of patients with missing data decreased from 81.8% to 67.1% (*P* < .001) for those with NSCLC, from 78.1% to 46.5% (*P* < .001) for those with breast cancer, and from 50.7% to 31.8% (*P* < .001) for those with prostate cancer (eFigure 5 in the Supplement). The changes in overall cancer stage are shown in eFigure 6 in the Supplement, and overall survival differences stratified by year of diagnosis are shown in eFigure 7 in the Supplement.

### Sensitivity Analysis

We repeated our analysis using variables of interest for which data were missing in 1% to 20% of patient records. Among those with NSCLC, 622 831 patients (52.0%) had missing data, and 575 918 patients (48.0%) had complete data; 2-year overall survival was 33.9% for patients with missing data and 43.5% for patients with complete data (*P* < .001). Among those with breast cancer, 1 481 729 patients (69.9%) had missing data, and 639 046 patients (30.1%) had complete data; 2-year overall survival was 92.4% for patients with missing data and 96.0% for patients with complete data (*P* < .001). Among those with prostate cancer, 700 523 patients (60.5%) had missing data, and 458 112 patients (39.5%) had complete data; 2-year overall survival was 91.7% for patients with missing data and 97.0% for patients without missing data (*P* < .001) (eFigure 8 in the Supplement).

Overall survival differences remained when we tested different thresholds using missing data cutoffs of either 1% to 5% or 5% to 30% (eFigure 9 in the Supplement). In the exploratory univariable analysis, the association between missing data and overall survival differed by individual variables (eTable 3 in the Supplement). Statistically significant variables included missing data overall and for clinical stage, laterality, tumor extension, regional nodes examined, sequence of surgery and radiotherapy, and facility type.

## Discussion

In a large national cancer registry, we found a high prevalence of missing data in the records of patients with the 3 most common cancer types. Missing data were associated with heterogeneous differences in overall survival and, in particular, with worse overall survival among patients with metastatic disease. The prevalence of missing data has marked implications for clinical care and research and suggests that there are substantial gaps in documenting and capturing data via the medical record for patients with cancer.

Significant differences were found with regard to demographic characteristics, tumor characteristics, and treatments received among patients with missing data vs complete data. Records with missing data were more prevalent among Black patients and patients from other racial and ethnic minority groups, which may reflect long-standing disparities in access to health care and cancer treatment.^24,25,26^ The records of patients with fewer comorbid conditions were also more frequently missing data, which may reflect less available documentation because of fewer medical visits. Patients with advanced-stage cancer were significantly more likely to have missing data. We hypothesize that this higher likelihood is associated with the increased complexity of care needed for patients with advanced-stage cancers, which may create increased difficulty in documenting and abstracting all data elements.^27^ The small survival differences among patients with early-stage breast and prostate cancers are reflective of this association with complexity, as definitive and adjuvant therapeutic management options in these settings are relatively less complex.

The study’s findings have several implications for clinical care. Missing data are clinically relevant because information that is important for treatment decision-making, such as cancer stage, may be incompletely documented. It is also plausible that while a clinical oncologist may have gathered adequate information (through interviewing and examining the patient, reviewing imaging, consulting with colleagues, or other means), the oncologist may not have documented this information in the medical record as text that could be abstracted through records review. In addition, given the multidisciplinary nature of cancer care, particularly for cases with increased complexity, communication of clinical information between oncologic specialists is often needed to determine the best course of treatment for a patient. However, when a patient’s care is fragmented between institutions, such communication often occurs primarily via the sharing of medical records. Therefore, missing data that cannot be abstracted from the medical record can have substantial implications for patients with fragmented courses of oncologic care. The high prevalence of missing data suggests that continued investment in data exchange standards remains an important step toward addressing the missing RWD problem for patients with cancer.^28,29^

These findings also have implications for RWD studies. Although incomplete documentation is ubiquitous in RWD sources, observational studies using large cancer registries often exclude patients with missing data, and the ways in which missing data are handled are inconsistently reported in the medical literature.^30,31^ Despite an increasing number of articles describing approaches for correcting missing data in observational studies, the practice of handling missing data among RWD sources has been slow to change.^32^ Recent systematic comparisons of registry studies and randomized clinical trials do not report concordant results.^7,9^ The lack of high-quality documentation is therefore a major obstacle when using modern RWD sources and can introduce substantial biases in research findings that rely on such data, potentially producing erroneous interpretations regarding real-world clinical outcomes. Within the NCDB, the relative importance of missing data for each individual variable was heterogeneous across cancer types. Variables providing information on staging and pathologic characteristics (such as overall and clinical stage, tumor extension, and pathologic lymph node evaluation) appeared to have highly statistically significant associations. Missing values for certain treatments (such as surgery and radiotherapy sequence) and demographic variables (such as facility type) were also statistically significant. Although quality control measures are used for the inclusion of information in the data registry, these findings reflect areas that require ongoing focus to improve the completeness of abstracted data.^33,34^

Although generating complete data for all patients is laborious and likely an untenable goal for large cancer registries given the number of patients and variables involved, there are a number of methods to address missing data within clinical data sets. These methods include the use of a missing data indicator or simple single value imputation, such as replacing missing values with the mean or mode based on nonmissing data, which may also introduce bias.^35^ Multiple imputation is less susceptible to bias compared with single imputation when data are missing at random, but multiple imputation depends on the appropriate modeling of each variable.^36,37^ Recent efforts employing machine-learning methods for imputation have indicated potential, but they often require substantial computational resources.^38,39^ Efforts to develop methods for capturing more complete data are ongoing. For example, greater adherence to structured data entry within the medical record may enable automatic abstraction of structured data elements.^40,41^ For unstructured data, natural language processing tools are being explored to capture information that would otherwise require substantial manual review for data abstraction.^42,43^

Data missingness itself may not be the reason for worse survival. The clinical explanations for survival differences associated with missing data are likely multifactorial. Significant differences were found in the distribution of cancer stage between patients with and without missing data. Within the NCDB, the distribution of cancer stage at diagnosis has also changed over time, which has previously been described.^44,45^ Differences in demographic characteristics, year of diagnosis, and treatments received are also associated factors. It is also likely that there are uncaptured confounders inherent to the observational nature of RWD studies. The decrease in missing data by year of diagnosis is reflective of improvements in coding standards and cancer registry quality over time. Our findings are consistent with those of other studies examining data missingness as a potential source of bias among RWD sources.^46,47,48,49^ Our results also align with previous analyses indicating substantial underascertainment of stage and treatment data within cancer-specific registries.^50,51,52^ Fragmented care is another plausible explanation for the association between missing data and survival among patients with cancer.^53^ Because registry data abstraction necessarily depends on information that is available within the patient record at the reporting facility, documentation quality, in particular, may have implications for patients with complex or fragmented disease courses.^54,55^

### Limitations

This study has several limitations. We examined overall survival and could not draw conclusions on other outcomes, such as toxic effects, disease recurrence, or factors associated with death. The data set within our study was obtained from an observational cancer registry, and there may be limitations in the data abstraction process that preclude complete capture of the medical record. Patient vital status (alive or dead) is reported to the NCDB from each institution. Given that the NCDB does not specify how this information is captured at each institution, there may be variability in the capture of overall survival data.^18,34^ However, all RWD sources likely have these limitations to a varying degree, and our analysis therefore should be interpreted as an exemplification of incomplete documentation within RWD sources in the oncology field.

Our study population is also heterogeneous. The patients’ cancer treatment protocols, including the receipt and sequence of local and systemic therapies, necessarily differ and do not reflect 1 specific clinical scenario. Nevertheless, overall survival differences between patients with missing and complete data remained despite adjustments for multiple tumor- and treatment-associated factors. In addition, the proportion of patients with missing data depends on the number of variables examined, as it is more difficult to have complete documentation for a larger number of data elements. Given that there are a large number of variables within the NCDB, we undertook an alternative analysis, in which we examined the variables of interest for which data were missing in 1% to 20% of records to identify patients with missing vs complete data. We also tested this assumption in a sensitivity analysis, which indicated that the overall survival difference persisted using either the 1% to 5% or 5% to 30% cutoff for missing data.

## Conclusions

The results of this study indicated that most patient records within a large cancer registry–based RWD source were subject to missing data. The prevalence of missing data that were unable to be ascertained from the medical record was associated with heterogeneous differences in overall survival and, in particular, worse survival among patients with metastatic cancer. Increasing the quality of documentation and adopting rigorous missing data correction methods are necessary to make optimal use of RWD for clinical advancements.

## Notes

Supplement. **eMethods.** The National Cancer Database **eTable 1.** Variables Used for All Years of Diagnosis and Disease Sites Included for Analysis **eTable 2.** Count of Patient Records Used for Comparison of Patient, Tumor, and Treatment Characteristics **eTable 3.** Univariable Cox Regression Analysis Using a Binary Indicator Variable of Whether Data Are Missing in Each Variable of Interest **eFigure 1.** Non–Small Cell Lung Cancer Overall Survival by Whether Data Are Missing in Variables of Interest and by Cancer Stage **eFigure 2.** Breast Cancer Overall Survival by Whether Data Are Missing in Variables of Interest and by Cancer Stage **eFigure 3.** Prostate Cancer Overall Survival by Whether Data Are Missing in Variables of Interest and by Cancer Stage **eFigure 4.** Overall Survival by Whether Data Are Missing in Variables of Interest and by Treatment Received **eFigure 5.** Proportion of Patients With Missing Data by Year of Diagnosis **eFigure 6.** Distribution of Cancer Stage by Year of Diagnosis **eFigure 7.** Overall Survival by Whether Data Are Missing in Variables of Interest and by Year of Diagnosis **eFigure 8.** Overall Survival by Complete vs Missing Data in Variables Missing in 1% to 20% of Patients **eFigure 9.** Sensitivity Analysis Varying Percentages of Missing Data
