# Supplementary material for: Prevalence of Missing Data in the National Cancer Database and Association With Overall Survival
Source: JAMA Netw Open. 2021 Mar 23;4(3):e211793. doi: 10.1001/jamanetworkopen.2021.1793 (PMC7988369; doi:10.1001/jamanetworkopen.2021.1793)
Supplement: Supplement. — eMethods. The National Cancer Database eTable 1. Variables Used for All Years of Diagnosis and Disease Sites Included for Analysis eTable 2. Count of Patient Records Used for Comparison of Patient, Tumor, and Treatment Characteristics eTable 3. Univariable Cox Regression Analysis Using a Binary Indicator Variable of Whether Data Are Missing in Each Variable of Interest eFigure 1. Non–Small Cell Lung Cancer Overall Survival by Whether Data Are Missing in Variables of Interest and by Cancer Stage eFigure 2. Breast Cancer Overall Survival by Whether Data Are Missing in Variables of Interest and by Cancer Stage eFigure 3. Prostate Cancer Overall Survival by Whether Data Are Missing in Variables of Interest and by Cancer Stage eFigure 4. Overall Survival by Whether Data Are Missing in Variables of Interest and by Treatment Received eFigure 5. Proportion of Patients With Missing Data by Year of Diagnosis eFigure 6. Distribution of Cancer Stage by Year of Diagnosis eFigure 7. Overall Survival by Whether Data Are Missing in Variables of Interest and by Year of Diagnosis eFigure 8. Overall Survival by Complete vs Missing Data in Variables Missing in 1% to 20% of Patients eFigure 9. Sensitivity Analysis Varying Percentages of Missing Data [file jamanetwopen-e211793-s001.pdf]

Supplementary Online Content

Yang DX, Khera R, Miccio JA, et al. Prevalence of missing data in the National Cancer Database and association with overall survival. *JAMA Netw Open*. 2021;4(3):e211793. doi:10.1001/jamanetworkopen.2021.1793

eMethods. The National Cancer Database

eTable 1. Variables Used for All Years of Diagnosis and Disease Sites Included for Analysis

eTable 2. Count of Patient Records Used for Comparison of Patient, Tumor, and Treatment Characteristics

eTable 3. Univariable Cox Regression Analysis Using a Binary Indicator Variable of Whether Data Are Missing in Each Variable of Interest

eFigure 1. Non–Small Cell Lung Cancer Overall Survival by Whether Data Are Missing in Variables of Interest and by Cancer Stage

eFigure 2. Breast Cancer Overall Survival by Whether Data Are Missing in Variables of Interest and by Cancer Stage

eFigure 3. Prostate Cancer Overall Survival by Whether Data Are Missing in Variables of Interest and by Cancer Stage

eFigure 4. Overall Survival by Whether Data Are Missing in Variables of Interest and by Treatment Received

eFigure 5. Proportion of Patients With Missing Data by Year of Diagnosis

eFigure 6. Distribution of Cancer Stage by Year of Diagnosis

eFigure 7. Overall Survival by Whether Data Are Missing in Variables of Interest and by Year of Diagnosis

eFigure 8. Overall Survival by Complete vs Missing Data in Variables Missing in 1% to 20% of Patients

eFigure 9. Sensitivity Analysis Varying Percentages of Missing Data

This supplementary material has been provided by the authors to give readers additional information about their work.

© 2021 Yang DX et al. *JAMA Network Open*.

eMethods. The National Cancer Database

Data reporting to the NCDB follows national registry coding standards and the recording of individual data elements have detailed specifications from the American College of Surgeons Commission on Cancer (CoC). Trained tumor registrars at individual cancer programs abstract specified data elements from patient records in accordance to registry data standards. If a data element is unable to be identified within the patient record, it may be recorded with a blank or unknown value following registry coding guidelines. Specific information abstracted include complex data elements such as comorbidity score, cancer stage, diagnostic procedures, treatment information including receipt of surgery, radiation, chemotherapy, among others.

Data elements in our study were categorized into facility and patient demographics (Demographics), tumor characteristics (Cancer Identification), cancer stage (Stage), cancer treatments (Treatment), and survival outcomes (Outcomes) variables based on the NCDB data dictionary. Two clinical oncologists reviewed all variables and excluded variables where blank data entry was allowed by the NCDB data dictionary and may not reflect incomplete clinical documentation. For example, days from diagnosis to chemotherapy was recorded as missing if the patient never received chemotherapy and therefore excluded from analysis. Furthermore, to be conservative, TNM pathologic staging were not considered missing if pathologic stage was not documented or a pathologic specimen was not collected. A final 63 variables of interest were identified to compare patients with and without completely documented data.

© 2021 Yang DX et al. *JAMA Network Open*.

eTable 1. Variables Used for All Years of Diagnosis and Disease Sites Included for Analysis

Category	Variable name	Missing in at least one patient record	Variable of interest
Demographics	AGE		
Demographics	CDCC_TOTAL_BEST		
Demographics	CROWFLY	X	X
Demographics	FACILITY_LOCATION_CD	X	X
Demographics	FACILITY_TYPE_CD	X	X
Demographics	INSURANCE_STATUS	X	X
Demographics	MED_INC_QUAR_00	X	X
Demographics	MED_INC_QUAR_12	X	X
Demographics	MED_INC_QUAR_16	X	X
Demographics	MEDICAID_EXPN_CODE		
Demographics	NO_HSD_QUAR_00	X	X
Demographics	NO_HSD_QUAR_12	X	X
Demographics	NO_HSD_QUAR_16	X	X
Demographics	PUF_CASE_ID		
Demographics	PUF_FACILITY_ID		
Demographics	PUF_MULT_SOURCE		
Demographics	RACE	X	X
Demographics	REFERENCE_DATE_FLAG		
Demographics	SEX		
Demographics	SPANISH_HISPANIC_ORIGIN	X	X
Demographics	UR_CD_03	X	X
Demographics	UR_CD_13	X	X
Cancer Identification	BEHAVIOR		
Cancer Identification	CLASS_OF_CASE		
Cancer Identification	DIAGNOSTIC_CONFIRMATION	X	X
Cancer Identification	GRADE	X	X
Cancer Identification	HISTOLOGY		
Cancer Identification	LATERALITY	X	X
Cancer Identification	PRIMARY_SITE		
Cancer Identification	REGIONAL_NODES_EXAMINED	X	X
Cancer Identification	REGIONAL_NODES_POSITIVE	X	X
Cancer Identification	SEQUENCE_NUMBER	X	X
Cancer Identification	YEAR_OF_DIAGNOSIS		
Stage	ANALYTIC_STAGE_GROUP	X	X
Stage	CS_EXTENSION	X	X
Stage	CS_METS_AT_DX	X	X
Stage	CS_METS_EVAL	X	X
Stage	CS_TUMOR_SIZEEXT_EVAL	X	X
Stage	CS_VERSION_LATEST	X	X
Stage	DX_STAGING_PROC_DAYS	X	

© 2021 Yang DX et al. *JAMA Network Open*.

Stage	RX_SUMM_DXSTG_PROC	X	X
Stage	TNM_CLIN_M	X	X
Stage	TNM_CLIN_N	X	X
Stage	TNM_CLIN_STAGE_GROUP	X	X
Stage	TNM_CLIN_T	X	X
Stage	TNM_EDITION_NUMBER	X	X
Stage	TNM_PATH_M	X	
Stage	TNM_PATH_N	X	
Stage	TNM_PATH_STAGE_GROUP	X	
Stage	TNM_PATH_T	X	
Stage	TUMOR_SIZE	X	X[a]
Treatment	DX_CHEMO_STARTED_DAYS	X	
Treatment	DX_DEFSURG_STARTED_DAYS	X	
Treatment	DX_HORMONE_STARTED_DAYS	X	
Treatment	DX_IMMUNO_STARTED_DAYS	X	
Treatment	DX_OTHER_STARTED_DAYS	X	
Treatment	DX_RAD_STARTED_DAYS	X	
Treatment	DX_RX_STARTED_DAYS	X	
Treatment	DX_SURG_STARTED_DAYS	X	
Treatment	DX_SYSTEMIC_STARTED_DAYS	X	
Treatment	PALLIATIVE_CARE	X	X
Treatment	PALLIATIVE_CARE_HOSP	X	X
Treatment	RAD_BOOST_DOSE_CGY	X	X
Treatment	RAD_BOOST_RX_MODALITY	X	X
Treatment	RAD_ELAPSED_RX_DAYS	X	X
Treatment	RAD_LOCATION_OF_RX	X	X
Treatment	RAD_NUM_TREAT_VOL	X	X
Treatment	RAD_REGIONAL_DOSE_CGY	X	X
Treatment	RAD_REGIONAL_RX_MODALITY	X	X
Treatment	RAD_TREAT_VOL	X	X
Treatment	READM_HOSP_30_DAYS	X	X
Treatment	REASON_FOR_NO_RADIATION	X	X
Treatment	REASON_FOR_NO_SURGERY	X	X
Treatment	RX_HOSP_CHEMO	X	X
Treatment	RX_HOSP_DXSTG_PROC	X	X
Treatment	RX_HOSP_HORMONE	X	X
Treatment	RX_HOSP_IMMUNOTHERAPY	X	X
Treatment	RX_HOSP_OTHER	X	X
Treatment	RX_HOSP_SURG_PRIM_SITE	X	X
Treatment	RX_SUMM_CHEMO	X	X
Treatment	RX_SUMM_HORMONE	X	X
Treatment	RX_SUMM_IMMUNOTHERAPY	X	X
Treatment	RX_SUMM_OTHER	X	X
Treatment	RX_SUMM_RADIATION	X	X

© 2021 Yang DX et al. *JAMA Network Open*.

Treatment	RX_SUMM_SCOPE_REG_LN_SUR	X	X
Treatment	RX_SUMM_SURG_OTH_REGDIS	X	X
Treatment	RX_SUMM_SURG_PRIM_SITE	X	X
Treatment	RX_SUMM_SURGICAL_MARGINS	X	
Treatment	RX_SUMM_SURGRAD_SEQ	X	X
Treatment	RX_SUMM_SYSTEMIC_SUR_SEQ	X	X
Treatment	RX_SUMM_TRNSPLNT_ENDO	X	X
Treatment	SURG_DISCHARGE_DAYS	X	
Outcomes	DX_LASTCONTACT_DEATH_MONTHS	X	
Outcomes	PUF_30_DAY_MORT_CD	X	
Outcomes	PUF_90_DAY_MORT_CD	X	
Outcomes	PUF_VITAL_STATUS		

[a] Tumor size was not used as a variable of interest for prostate cancer given it is not routinely reported in clinical practice

© 2021 Yang DX et al. *JAMA Network Open*.

eTable 2. Count of Patient Records Used for Comparison of Patient, Tumor, and Treatment Characteristics

A. Non-small cell lung cancer

	Records used		Records not used[a]	
	**Complete data**	**Missing data**	**Complete data**	**Missing data**
Age at Diagnosis	347,454	851,295	0	0
Sex	347,454	851,295	0	0
Race	347,454	842,127	0	9,168
Hispanic Ethnicity	347,454	784,015	0	67,280
Charlson-Deyo Score	347,454	851,295	0	0
Insurance	347,454	827,936	0	23,359
Facility Type	347,454	843,657	0	7,638
Year of Diagnosis	347,454	851,295	0	0
Overall Stage	347,395	776,500	59	74,795
Tumor Size	346,962	626,110	492	225,185
Lymph Nodes Involved	336,910	692,475	10,544	158,820
Distant Metastasis	347,454	815,377	0	35,918
Surgery (Primary Site)	347,454	847,710	0	3,585
Radiation	347,454	840,924	0	10,371
Chemotherapy	347,454	811,051	0	40,244

B. Breast cancer

	Records used		Records not used	
	**Complete data**	**Missing data**	**Complete data**	**Missing data**
Age at Diagnosis	959,679	1,161,096	0	0
Sex	959,679	1,161,096	0	0
Race	959,679	1,138,156	0	22,940
Hispanic Ethnicity	959,679	1,049,841	0	111,255
Charlson-Deyo Score	959,679	1,161,096	0	0
Insurance	959,679	1,122,489	0	38,607
Facility Type	959,679	1,067,375	0	93,721
Year of Diagnosis	959,679	1,161,096	0	0
Overall Stage	959,361	1,089,661	318	71,435
Tumor Size	956,593	954,416	3,086	206,680
Lymph Nodes Involved	945,511	991,196	14,168	169,900
Distant Metastasis	959,361	1,083,580	318	77,516
Surgery (Primary Site)	959,679	1,156,603	0	4,493
Radiation	959,679	1,139,214	0	21,882
Chemotherapy	959,679	1,093,054	0	68,042

© 2021 Yang DX et al. *JAMA Network Open*.

Hormonal Therapy	959,679	1,066,418	0	94,678

C. Prostate cancer

	Records used		Records not used	
	**Complete data**	**Missing data**	**Complete data**	**Missing data**
Age at Diagnosis	698,468	460,167	0	0
Sex	698,468	460,167	0	0
Race	698,468	441,710	0	18,457
Hispanic Ethnicity	698,468	386,668	0	73,499
Charlson-Deyo Score	698,468	460,167	0	0
Insurance	698,468	436,545	0	23,622
Facility Type	698,468	459,296	0	871
Year of Diagnosis	698,468	460,167	0	0
Overall Stage	698,430	403,550	38	56,617
Lymph Nodes Involved	668,940	356,173	29,528	103,994
Distant Metastasis	698,429	417,866	39	42,301
Surgery (Primary Site)	698,468	456,891	0	3,276
Radiation	698,468	449,371	0	10,796
Chemotherapy	698,468	423,743	0	36,424
Hormonal Therapy	698,468	419,513	0	40,654

[a] Patient records not used that have complete data are due to recoding of the variable for comparison.

© 2021 Yang DX et al. *JAMA Network Open*.

eTable 3. Univariable Cox Regression Analysis Using a Binary Indicator Variable of Whether Data Are Missing in Each Variable of Interest

A. Non-small cell lung cancer

Variable	Coefficient	P-Value
TUMOR_SIZE	0.75	<0.001
LATERALITY	0.70	<0.001
CS_EXTENSION	0.64	<0.001
CS_METS_AT_DX	0.61	<0.001
RX_SUMM_SURGRAD_SEQ	0.58	<0.001
PALLIATIVE_CARE_HOSP	0.55	<0.001
GRADE	0.54	<0.001
RAD_LOCATION_OF_RX	0.51	<0.001
RAD_TREAT_VOL	0.48	<0.001
RAD_REGIONAL_RX_MODALITY	0.48	<0.001
REASON_FOR_NO_RADIATION	0.44	<0.001
RAD_BOOST_RX_MODALITY	0.39	<0.001
RAD_BOOST_DOSE_CGY	0.38	<0.001
CS_TUMOR_SIZEEXT_EVAL	0.33	<0.001
RAD_ELAPSED_RX_DAYS	0.33	<0.001
RAD_REGIONAL_DOSE_CGY	0.31	<0.001
REGIONAL_NODES_POSITIVE	0.31	<0.001
RAD_NUM_TREAT_VOL	0.30	<0.001
TNM_CLIN_STAGE_GROUP	-0.40	<0.001
FACILITY_TYPE_CD	-0.61	<0.001
FACILITY_LOCATION_CD	-0.61	<0.001
TNM_EDITION_NUMBER	-1.00	<0.001
REGIONAL_NODES_EXAMINED	-0.18	<0.001
REASON_FOR_NO_SURGERY	0.26	<0.001
RX_HOSP_IMMUNOTHERAPY	-0.51	<0.001
RX_SUMM_IMMUNOTHERAPY	-0.50	<0.001
RX_HOSP_HORMONE	-0.49	<0.001
RX_HOSP_CHEMO	-0.33	<0.001
TNM_CLIN_M	-0.20	<0.001
RX_SUMM_SYSTEMIC_SUR_SEQ	-0.24	<0.001
RX_SUMM_RADIATION	-0.33	<0.001
RX_SUMM_CHEMO	-0.15	<0.001
RX_SUMM_HORMONE	-0.17	<0.001
RX_SUMM_TRNSPLNT_ENDO	-0.28	<0.001
DIAGNOSTIC_CONFIRMATION	0.44	<0.001
RX_SUMM_SURG_OTH_REGDIS	-0.29	<0.001
CS_METS_EVAL	0.13	<0.001
SPANISH_HISPANIC_ORIGIN	0.08	<0.001
ANALYTIC_STAGE_GROUP	0.07	<0.001
PALLIATIVE_CARE	-0.16	<0.001
READM_HOSP_30_DAYS	-0.07	<0.001
RX_SUMM_DXSTG_PROC	-0.10	<0.001
NO_HSD_QUAR_00	-0.05	<0.001

© 2021 Yang DX et al. *JAMA Network Open*.

MED_INC_QUAR_00	-0.05	<0.001
UR_CD_13	-0.04	<0.001
UR_CD_03	-0.04	<0.001
RX_HOSP_SURG_PRIM_SITE	0.17	<0.001
INSURANCE_STATUS	0.03	<0.001
RX_SUMM_SCOPE_REG_LN_SUR	-0.04	<0.001
RX_SUMM_SURG_PRIM_SITE	-0.07	<0.001
MED_INC_QUAR_16	0.03	0.002
RACE	-0.03	0.026
NO_HSD_QUAR_16	0.02	0.082
TNM_CLIN_N	-0.01	0.135
TNM_CLIN_T	-0.01	0.263
MED_INC_QUAR_12	0.02	0.295
RX_HOSP_DXSTG_PROC	0.01	0.396
SEQUENCE_NUMBER	0.04	0.683
NO_HSD_QUAR_12	0.01	0.748
RX_HOSP_OTHER	-0.02	0.857
RX_SUMM_OTHER	-0.01	0.870
CROWFLY	0.00	0.889
CS_VERSION_LATEST	-22.00	0.999

B. Breast cancer

Variable	Coefficient	P-Value
LATERALITY	1.91	<0.001
CS_EXTENSION	1.61	<0.001
REASON_FOR_NO_SURGERY	0.91	<0.001
CS_METS_AT_DX	0.86	<0.001
REGIONAL_NODES_POSITIVE	0.79	<0.001
CS_TUMOR_SIZEEXT_EVAL	0.73	<0.001
RX_SUMM_SURGRAD_SEQ	0.58	<0.001
CS_METS_EVAL	0.54	<0.001
REGIONAL_NODES_EXAMINED	0.50	<0.001
RAD_LOCATION_OF_RX	0.46	<0.001
RAD_TREAT_VOL	0.43	<0.001
REASON_FOR_NO_RADIATION	0.37	<0.001
ANALYTIC_STAGE_GROUP	0.37	<0.001
RAD_REGIONAL_RX_MODALITY	0.41	<0.001
GRADE	0.17	<0.001
FACILITY_TYPE_CD	-0.31	<0.001
FACILITY_LOCATION_CD	-0.31	<0.001
TNM_CLIN_STAGE_GROUP	-0.13	<0.001
RX_SUMM_SYSTEMIC_SUR_SEQ	-0.29	<0.001
DIAGNOSTIC_CONFIRMATION	1.42	<0.001
PALLIATIVE_CARE_HOSP	0.44	<0.001
RX_SUMM_IMMUNOTHERAPY	-0.30	<0.001
RX_HOSP_IMMUNOTHERAPY	-0.31	<0.001
RX_HOSP_HORMONE	-0.23	<0.001
RX_SUMM_HORMONE	-0.16	<0.001

© 2021 Yang DX et al. *JAMA Network Open*.

TNM_EDITION_NUMBER	0.62	<0.001
RX_HOSP_SURG_PRIM_SITE	0.96	<0.001
RX_SUMM_TRNSPLNT_ENDO	-0.25	<0.001
RAD_NUM_TREAT_VOL	-0.12	<0.001
RX_SUMM_SURG_PRIM_SITE	0.56	<0.001
RACE	-0.30	<0.001
RAD_BOOST_RX_MODALITY	0.11	<0.001
TNM_CLIN_T	-0.17	<0.001
RX_SUMM_CHEMO	-0.11	<0.001
RX_HOSP_CHEMO	-0.15	<0.001
RX_SUMM_DXSTG_PROC	-0.21	<0.001
NO_HSD_QUAR_00	-0.09	<0.001
MED_INC_QUAR_00	-0.09	<0.001
RX_SUMM_SCOPE_REG_LN_SUR	0.17	<0.001
READM_HOSP_30_DAYS	-0.08	<0.001
UR_CD_13	-0.07	<0.001
UR_CD_03	-0.07	<0.001
RAD_BOOST_DOSE_CGY	0.06	<0.001
INSURANCE_STATUS	0.07	<0.001
TNM_CLIN_N	0.09	<0.001
RX_HOSP_DXSTG_PROC	0.15	<0.001
RAD_ELAPSED_RX_DAYS	0.03	<0.001
RAD_REGIONAL_DOSE_CGY	-0.03	<0.001
PALLIATIVE_CARE	0.07	0.009
TUMOR_SIZE	0.01	0.013
MED_INC_QUAR_16	0.03	0.026
CS_VERSION_LATEST	1.92	0.055
SPANISH_HISPANIC_ORIGIN	0.01	0.098
NO_HSD_QUAR_16	0.02	0.121
MED_INC_QUAR_12	0.04	0.169
RX_SUMM_SURG_OTH_REGDIS	0.03	0.173
RX_HOSP_OTHER	0.15	0.262
SEQUENCE_NUMBER	0.26	0.263
RX_SUMM_OTHER	0.13	0.312
RX_SUMM_RADIATION	0.01	0.705
NO_HSD_QUAR_12	0.01	0.782
TNM_CLIN_M	0.00	0.898
CROWFLY	0.00	0.978

C. Prostate cancer

**Variable**	**Coefficient**	**P-Value**
GRADE	1.40	<0.001
TNM_CLIN_T	0.76	<0.001
CS_METS_AT_DX	0.85	<0.001
CS_EXTENSION	1.69	<0.001
CS_TUMOR_SIZEEXT_EVAL	0.92	<0.001
REGIONAL_NODES_POSITIVE	0.57	<0.001
ANALYTIC_STAGE_GROUP	0.30	<0.001

© 2021 Yang DX et al. *JAMA Network Open*.

TNM_CLIN_N	0.57	<0.001
RACE	-0.52	<0.001
TNM_EDITION_NUMBER	1.07	<0.001
REASON_FOR_NO_SURGERY	0.36	<0.001
RX_HOSP_DXSTG_PROC	0.72	<0.001
RX_SUMM_HORMONE	-0.24	<0.001
REGIONAL_NODES_EXAMINED	0.23	<0.001
RX_HOSP_HORMONE	-0.30	<0.001
CS_METS_EVAL	0.20	<0.001
DIAGNOSTIC_CONFIRMATION	0.59	<0.001
REASON_FOR_NO_RADIATION	0.16	<0.001
RX_SUMM_DXSTG_PROC	0.29	<0.001
RX_SUMM_SURG_OTH_REGDIS	-0.30	<0.001
PALLIATIVE_CARE	-0.33	<0.001
RX_HOSP_IMMUNOTHERAPY	-0.19	<0.001
RX_SUMM_IMMUNOTHERAPY	-0.19	<0.001
RX_SUMM_TRNSPLNT_ENDO	-0.16	<0.001
SPANISH_HISPANIC_ORIGIN	-0.07	<0.001
RX_HOSP_SURG_PRIM_SITE	0.54	<0.001
TNM_CLIN_STAGE_GROUP	0.05	<0.001
NO_HSD_QUAR_00	-0.10	<0.001
RAD_REGIONAL_DOSE_CGY	0.11	<0.001
MED_INC_QUAR_00	-0.10	<0.001
RX_SUMM_CHEMO	-0.10	<0.001
RX_SUMM_SURGRAD_SEQ	0.15	<0.001
RAD_BOOST_RX_MODALITY	0.11	<0.001
RAD_BOOST_DOSE_CGY	0.10	<0.001
RAD_TREAT_VOL	0.11	<0.001
RAD_ELAPSED_RX_DAYS	0.08	<0.001
RAD_REGIONAL_RX_MODALITY	0.10	<0.001
UR_CD_03	-0.08	<0.001
UR_CD_13	-0.08	<0.001
RX_SUMM_RADIATION	-0.12	<0.001
RAD_LOCATION_OF_RX	0.09	<0.001
RX_SUMM_SYSTEMIC_SUR_SEQ	-0.06	<0.001
RAD_NUM_TREAT_VOL	0.05	<0.001
TNM_CLIN_M	0.05	<0.001
FACILITY_TYPE_CD	-0.33	0.001
FACILITY_LOCATION_CD	-0.33	0.001
RX_SUMM_SCOPE_REG_LN_SUR	-0.07	0.008
READM_HOSP_30_DAYS	0.04	0.027
MED_INC_QUAR_12	0.07	0.064
NO_HSD_QUAR_12	0.07	0.098
RX_HOSP_CHEMO	-0.03	0.099
INSURANCE_STATUS	0.02	0.123
RX_SUMM_SURG_PRIM_SITE	0.06	0.161
RX_SUMM_OTHER	0.22	0.217

© 2021 Yang DX et al. *JAMA Network Open*.

RX_HOSP_OTHER	0.22	0.228
PALLIATIVE_CARE_HOSP	-0.04	0.235
SEQUENCE_NUMBER	0.34	0.241
CROWFLY	0.06	0.244
NO_HSD_QUAR_16	-0.01	0.673
MED_INC_QUAR_16	0.00	0.821
CS_VERSION_LATEST	-21.00	0.999
LATERALITY[a]	-	-

[a] There were no missing values for laterality for prostate cancer. Prostate is not a paired site.

© 2021 Yang DX et al. *JAMA Network Open*.

eFigure 1. Non–Small Cell Lung Cancer Overall Survival by Whether Data Are Missing in Variables of Interest and by Cancer Stage

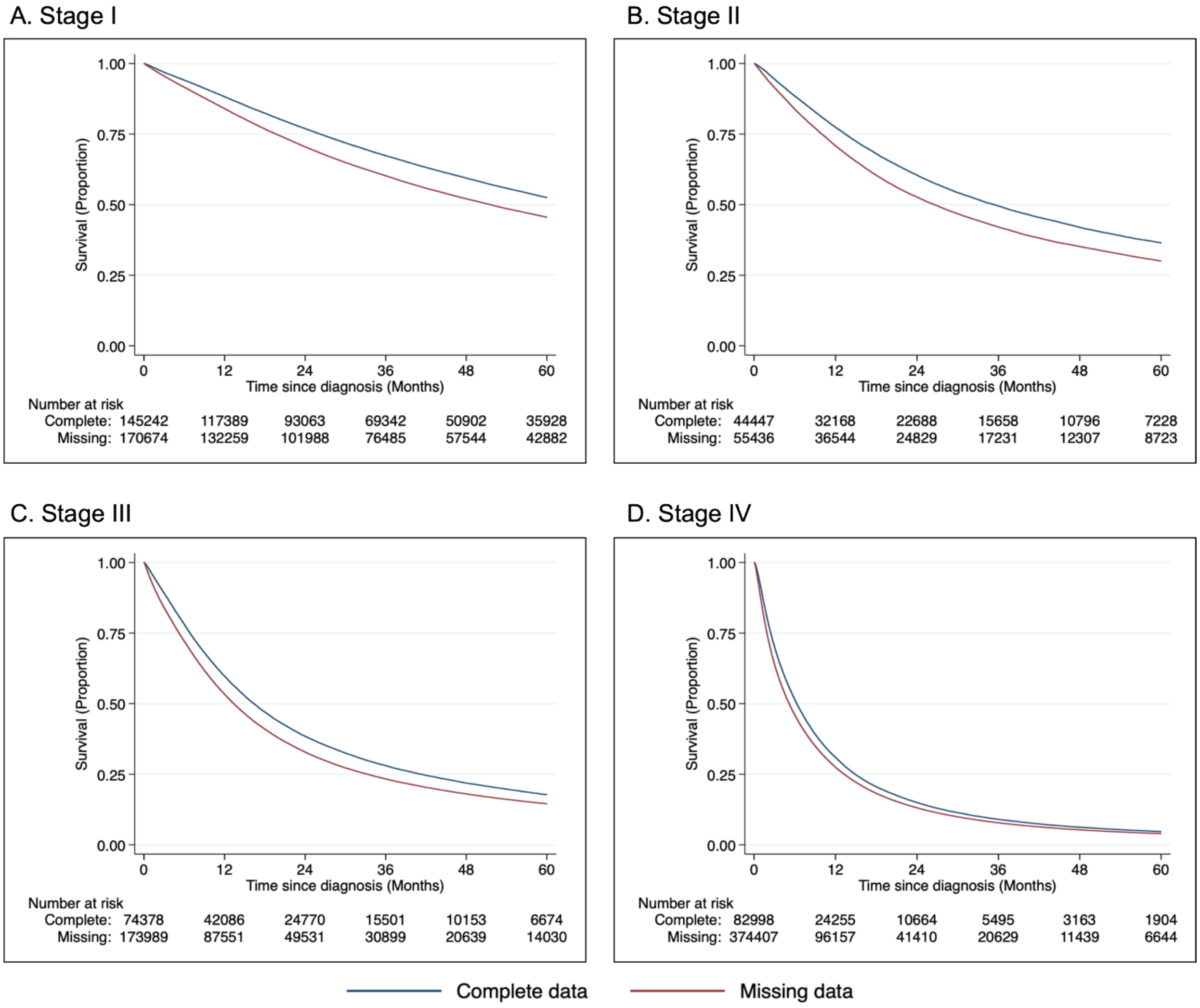

Analytic group stages 0 and I were combined for stage I subgroup analysis for non-small cell lung cancer

© 2021 Yang DX et al. *JAMA Network Open*.

eFigure 2. Breast Cancer Overall Survival by Whether Data Are Missing in Variables of Interest and by Cancer Stage

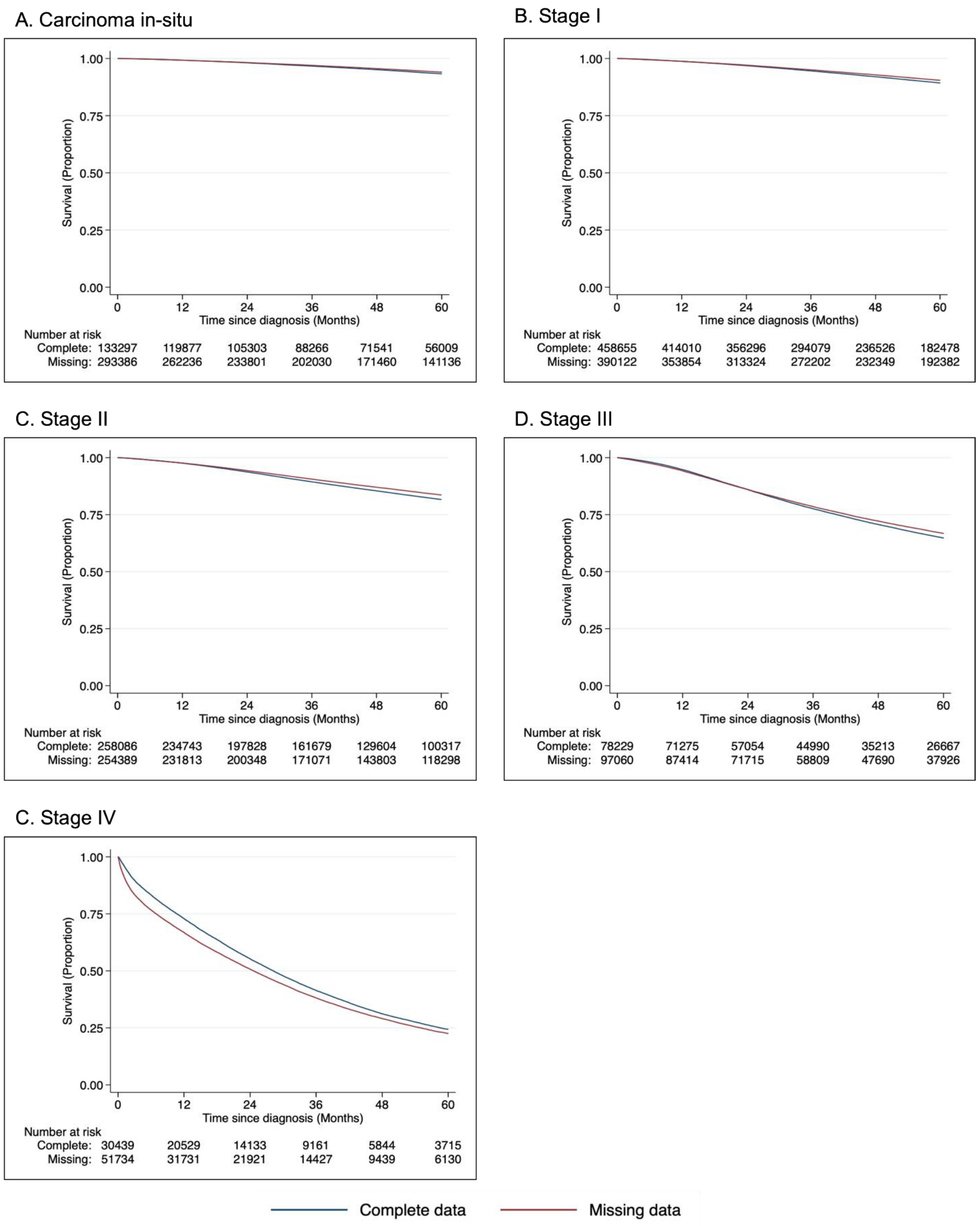

© 2021 Yang DX et al. *JAMA Network Open*.

eFigure 3. Prostate Cancer Overall Survival by Whether Data Are Missing in Variables of Interest and by Cancer Stage

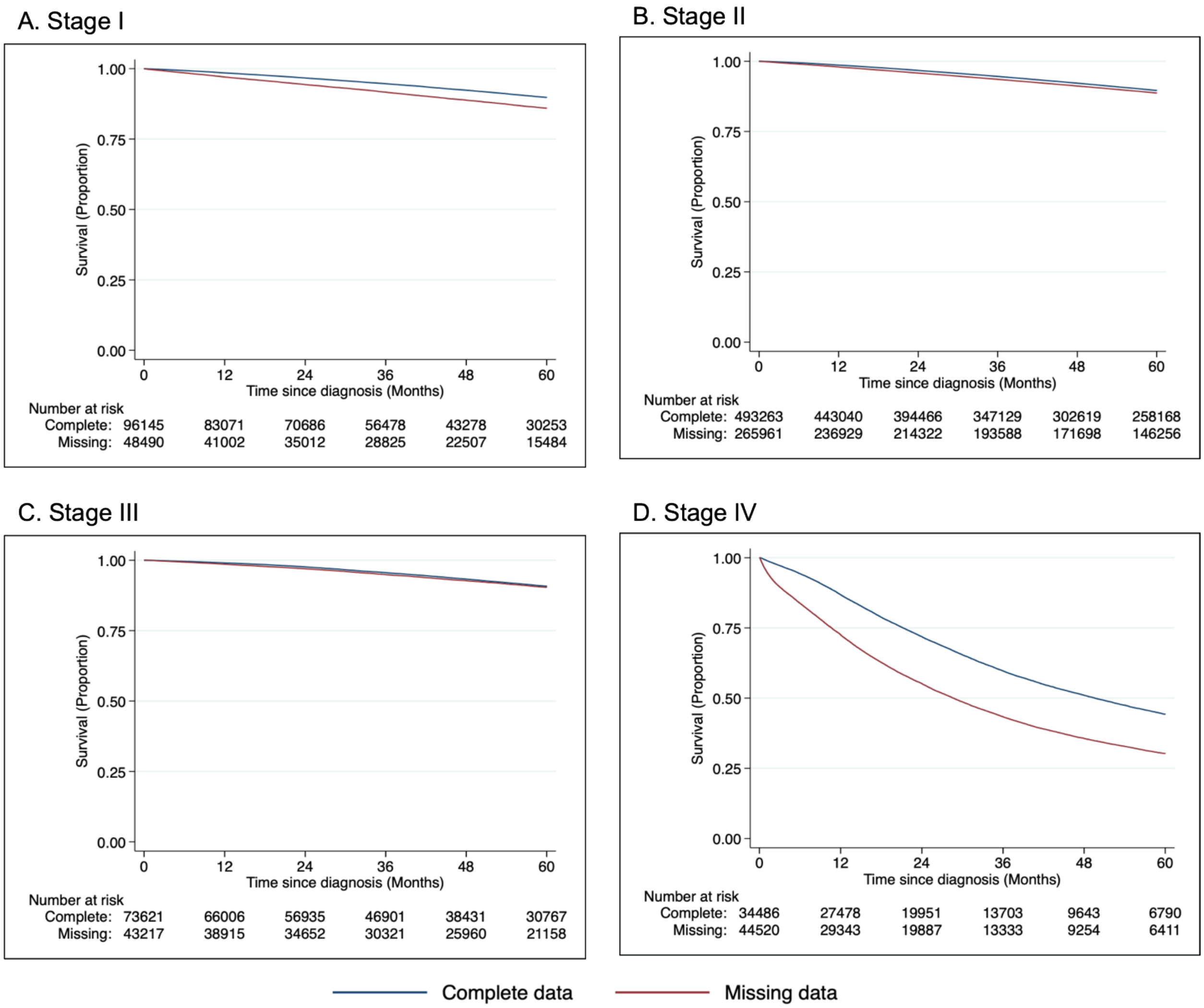

© 2021 Yang DX et al. *JAMA Network Open*.

eFigure 4. Overall Survival by Whether Data Are Missing in Variables of Interest and by Treatment Received

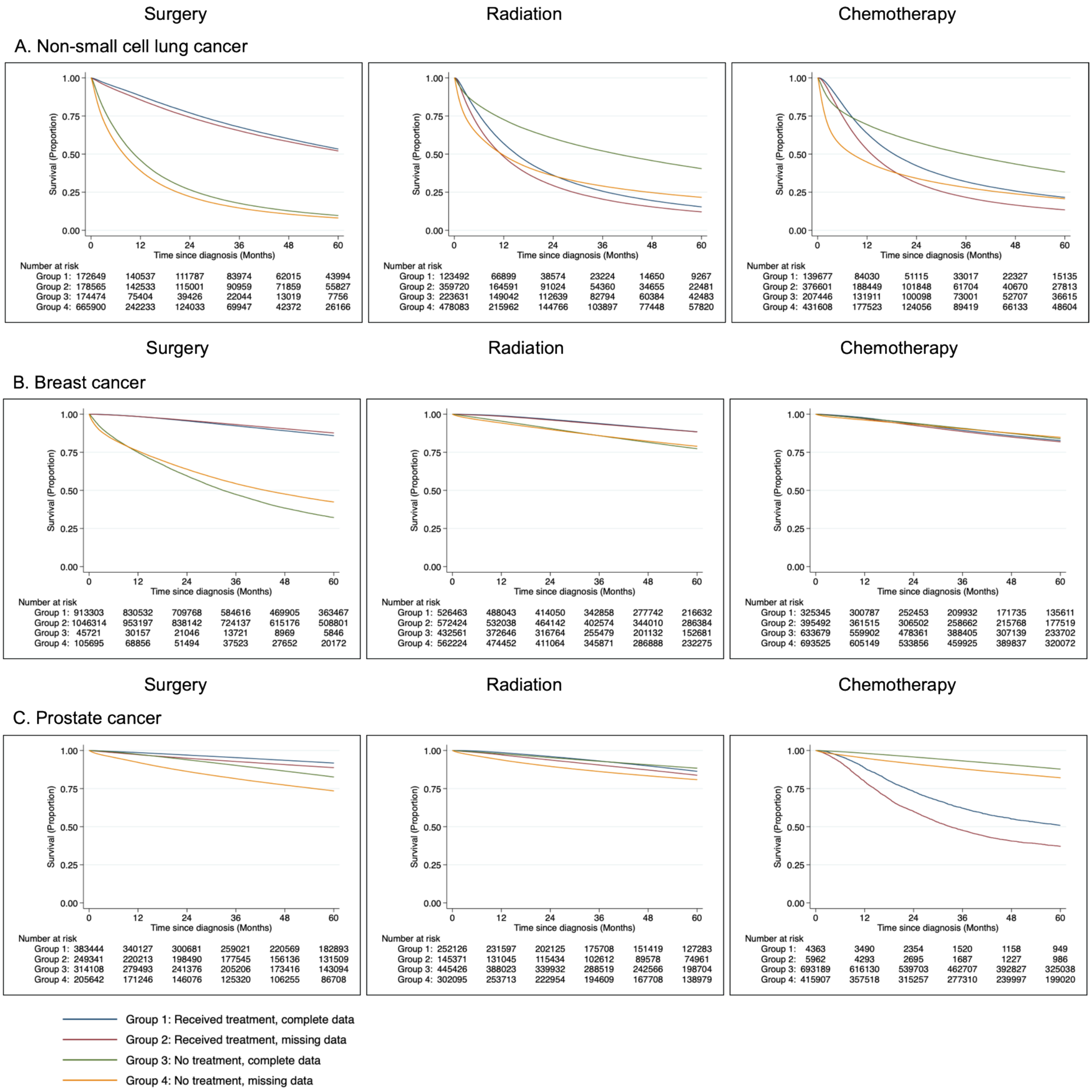

© 2021 Yang DX et al. *JAMA Network Open*.

eFigure 5. Proportion of Patients With Missing Data by Year of Diagnosis

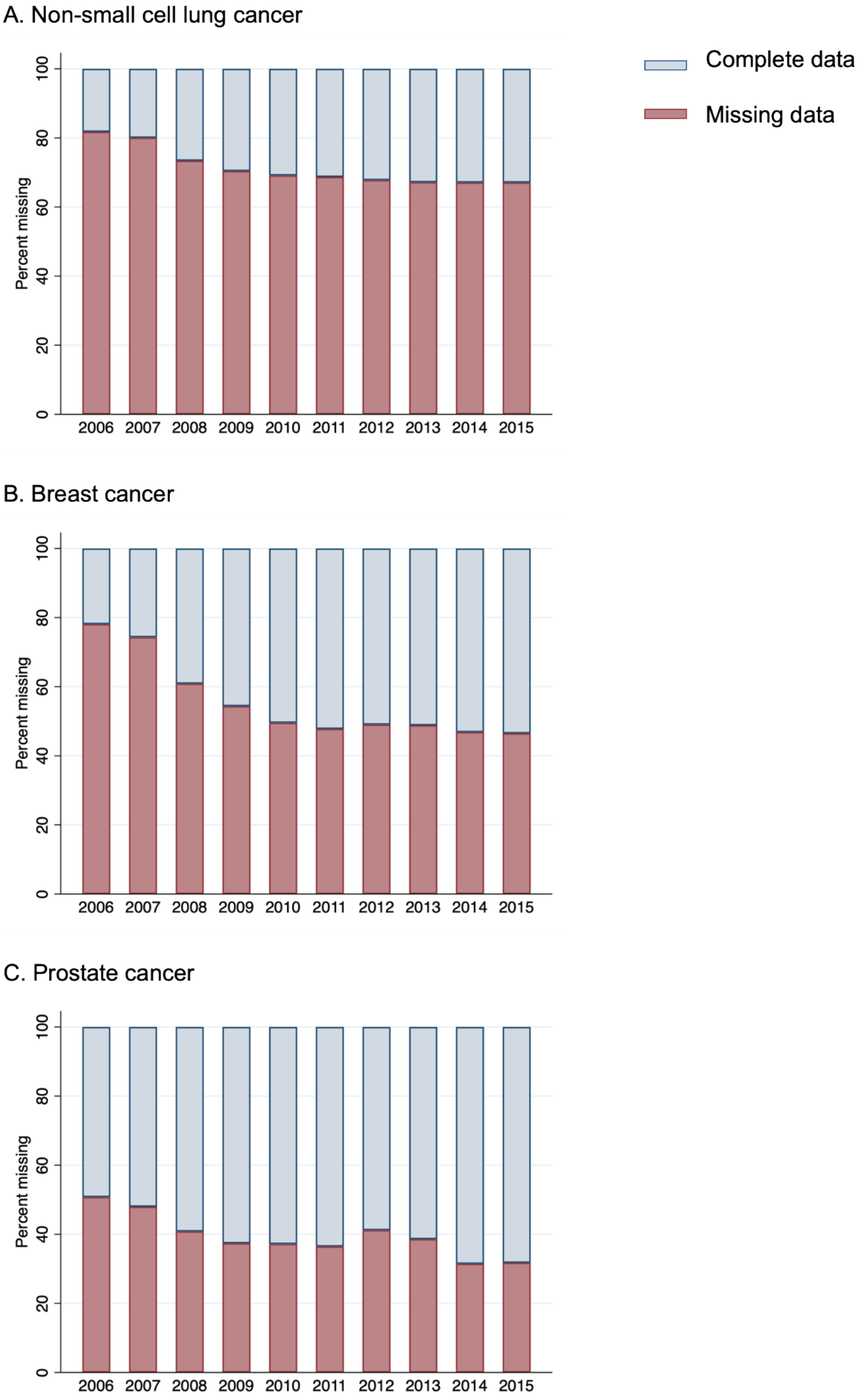

© 2021 Yang DX et al. *JAMA Network Open*.

eFigure 6. Distribution of Cancer Stage by Year of Diagnosis

A. Non-small cell lung cancer

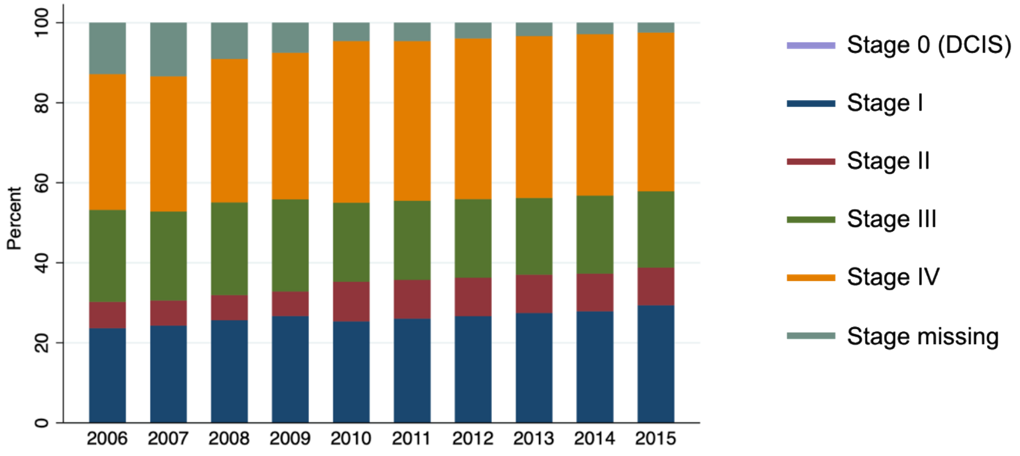

B. Breast cancer

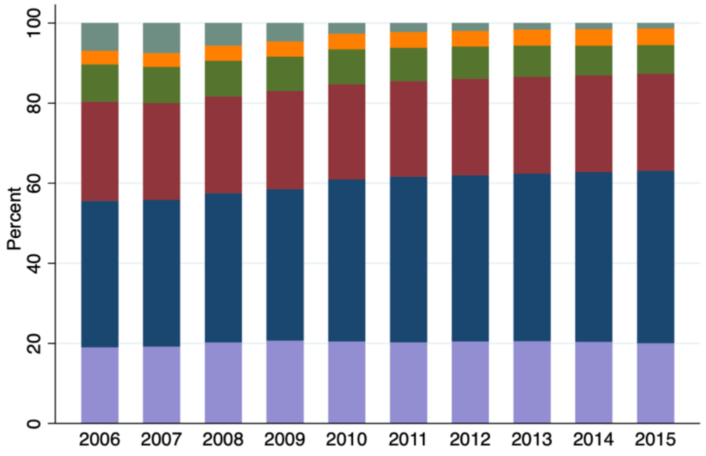

C. Prostate cancer

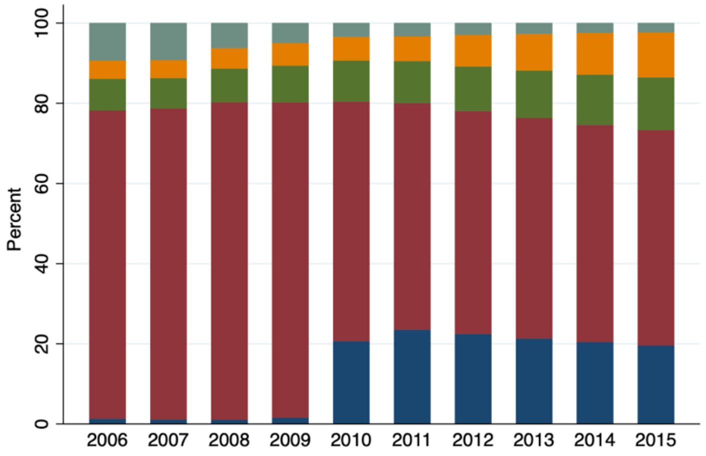

© 2021 Yang DX et al. *JAMA Network Open*.

eFigure 7. Overall Survival by Whether Data Are Missing in Variables of Interest and by Year of Diagnosis

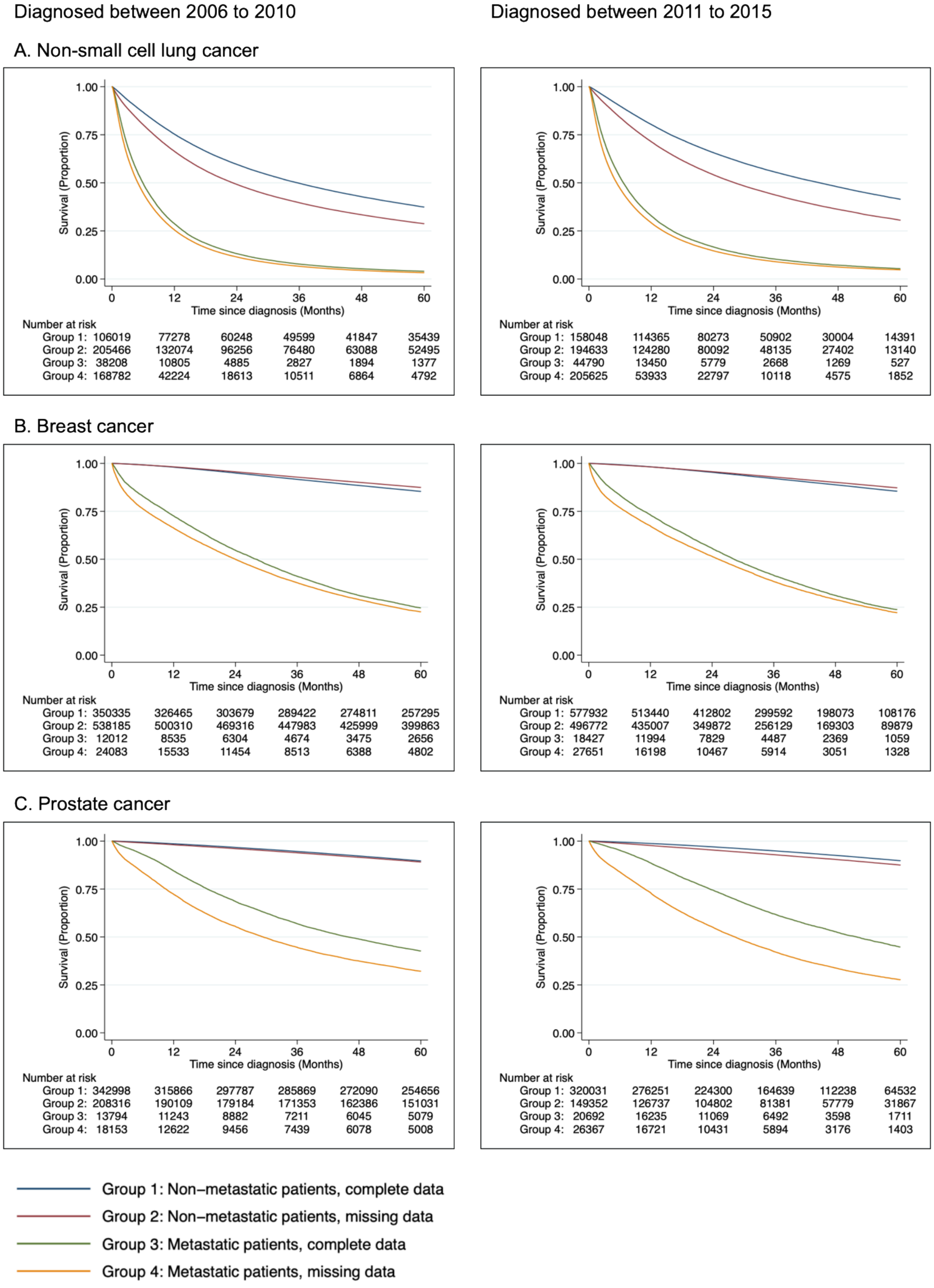

© 2021 Yang DX et al. *JAMA Network Open*.

eFigure 8. Overall Survival by Complete vs Missing Data in Variables Missing in 1% to 20% of Patients

A. Non-small cell lung cancer

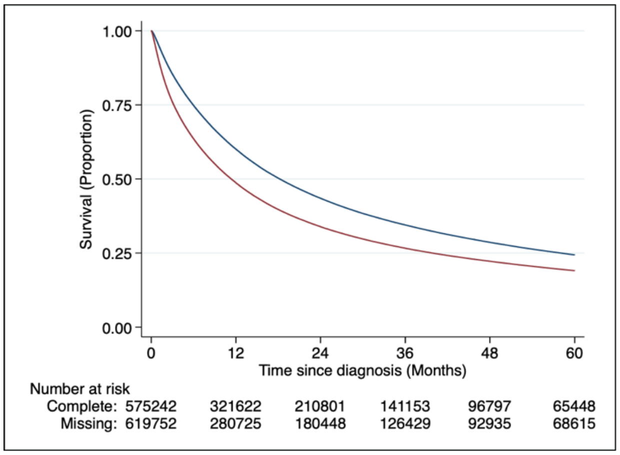

B. Breast cancer

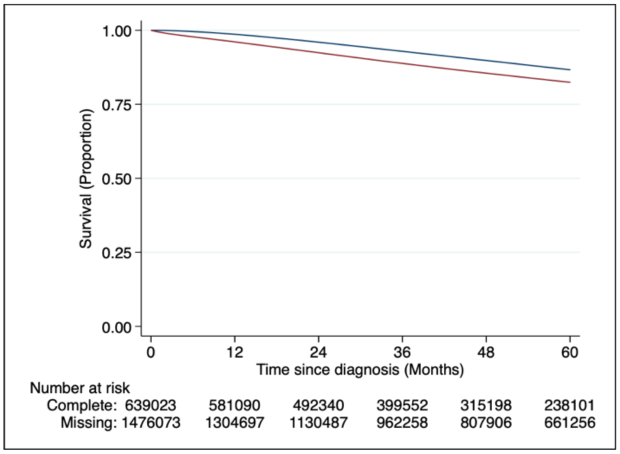

C. Prostate cancer

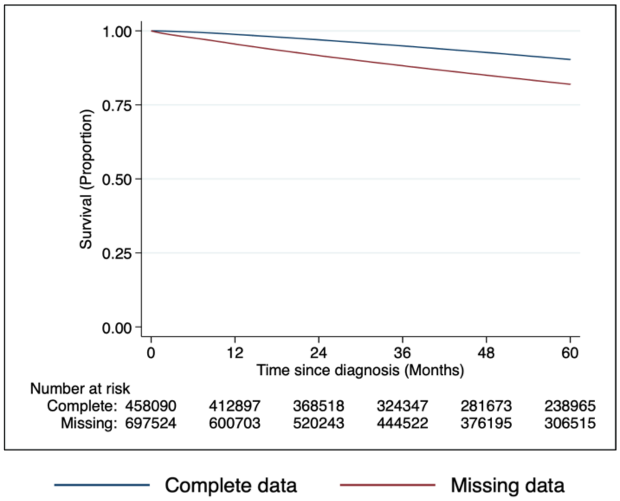

© 2021 Yang DX et al. *JAMA Network Open*.

eFigure 9. Sensitivity Analysis Varying Percentages of Missing Data

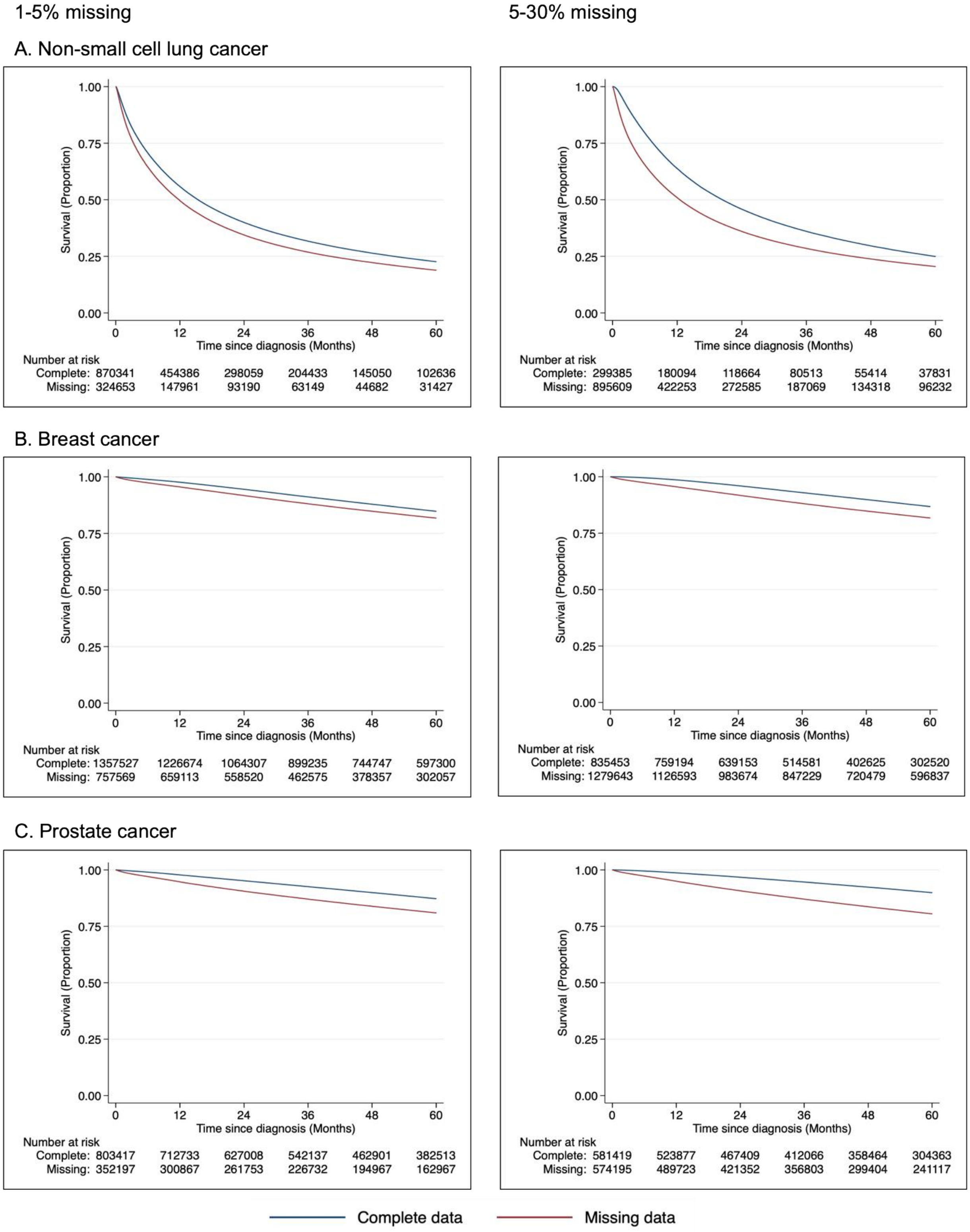

© 2021 Yang DX et al. *JAMA Network Open*.
